# Experimental and FEM Analysis of Slab Structures Reinforced with Tubular Reinforcement

**DOI:** 10.3390/ma18102369

**Published:** 2025-05-20

**Authors:** Tae-Hee Lee, Gun Jung, Taehoon Han, Jang-Ho Jay Kim

**Affiliations:** 1 School of Civil and Environmental Engineering, Yonsei University, 50, Yonsei-ro, Seodaemun-gu, Seoul 03722, Republic of Korea; saintlth@yonsei.ac.kr; 2 DL E&C., 26, Tongil-ro, Jongno-gu, Seoul 03181, Republic of Korea; junggun@dlenc.co.kr; 3 KONES., 65, Myeongdal-ro, Seocho-gu, Seoul 06667, Republic of Korea; sg02520@kones21.com

**Keywords:** flexural behavior, tubular reinforcement, slab structures, finite element analysis

## Abstract

This study investigates the structural behavior of reinforced concrete slabs and culverts using newly developed tubular rebars as a replacement for conventional deformed rebars. Tubular rebars, which are approximately 50% lighter and exhibit twice the tensile strength of standard deformed rebars, were evaluated through experimental tests and finite element analysis (FEA). Results showed that tubular rebars achieved up to 44.46% higher yield strength and up to 25.31% higher ultimate strength in statically determinate slabs compared to conventional rebars, though with reduced ductility. In statically indeterminate configurations such as fixed slabs and box culverts, the ductility performance improved significantly, with ductility index differences reduced to less than 3%. Hybrid reinforcement combining tubular and deformed rebars also enhanced performance, especially in compression zones. These findings demonstrate that tubular rebars can be a sustainable and structurally efficient alternative to conventional reinforcement when deflection control is ensured.

## 1. Introduction

Due to the increasing concentration of urban populations and the limited availability of land, modern structures are becoming taller, larger, and more space-efficient. Consequently, the demand for construction materials, such as rebar and cement, continues to rise. Over the past five years, global crude steel production, a primary material for rebar, has increased by an average of 2.3% annually [1]. Future steel demand is projected to grow between 1.4% and 3.3% per year [2,3,4]. However, steel production incurs significant environmental costs, particularly through greenhouse gas (GHG) emissions. On average, the production of crude steel generates 1.91 tonnes of CO_2_ per tonne of steel, accounting for approximately 7–9% of global fossil fuel emissions [5]. As a result, the construction industry is actively seeking ways to reduce CO_2_ emissions.

While many studies have focused on reducing the carbon footprint of concrete (particularly cement) through material substitution or admixtures [6,7], comparatively fewer studies have explored the sustainability potential of reinforcement optimization [8]. Since steel reinforcement also accounts for a substantial portion of embodied energy in reinforced concrete structures, improving the efficiency and mechanical performance of rebar can lead to meaningful reductions in total material use and environmental impact. Therefore, this study focuses on the sustainability of reinforcement rather than concrete as a strategic approach to material reduction.

One proposed solution in Korea involves replacing traditional deformed rebar with tubular rebar. The newly developed STG800 tubular rebar is a high-strength material that requires less raw material than conventional deformed rebar, thereby reducing GHG emissions. This tubular rebar has a hollow section, a yield strength of 800 MPa, and the same diameter as deformed rebar. It is also approximately 5–10% cheaper and 50% lighter per meter than conventional deformed rebar. An et al. evaluated the use of a tubular rebar net in a landslide protection wall, demonstrating a 20% improvement in bending performance, confirming its applicability to real structures [9].

High-strength rebars, including tubular rebars, reduce the overall amount of reinforcement needed in structures. Rautenberg et al. confirmed that using rebars with a strength exceeding 80 ksi (550 MPa) allows for a reduction in the quantity of main reinforcement [10]. However, while high-strength rebars offer durability and enhanced mechanical properties, they often exhibit reduced ductility [11]. Since reinforced concrete (RC) members require adequate ductility in addition to strength, design standards in various countries impose limitations on the maximum yield strength of rebar [12,13,14,15]. Shahrooz et al. suggested modifying strain limits and restricting service load stress to 60% of the yield strength to ensure comparable curvature behavior in bending members using high-strength rebars [16]. Thus, securing adequate ductility is essential for the effective application of high-strength rebars.

Tubular rebars, in particular, are expected to exhibit lower ductility than conventional deformed rebars. While deformed rebars resist deflection across their entire cross-section, tubular rebars cannot fully resist deflection due to their hollow geometry. Therefore, structural deflection control is crucial for their application [17,18]. Although previous studies have explored the mechanical properties of tubular rebar, including bond behavior and fire resistance, research on its practical application in real-world structures remains limited.

Therefore, this study aims to investigate the structural performance of reinforced concrete members using tubular rebars through both experimental and numerical approaches. The research gap lies in the lack of data on the application of tubular reinforcement in full-scale elements under realistic conditions, particularly regarding flexural behavior and ductility. The remainder of this paper is organized as follows: Section 2 presents the experimental program, Section 3 describes the numerical simulation and comparison with the experimental results, Section 4 provides additional experimental verification through a bending test on a box culvert, a representative statically indeterminate structure, Section 5 discusses the implications and contributions of the findings, and Section 6 concludes the paper.

## 2. Experimental Evaluation of Slab Specimen

### 2.1. Material

Specimens were manufactured using the following materials to evaluate the performance when the newly developed tubular rebar replaced the deformed rebar. The same concrete mix proportion was used for all specimens, and the existing deformed rebar and tubular rebar were used for reinforcement in concrete.

#### 2.1.1. Concrete Mixture

In this study, concrete manufactured in a ready-mixed concrete factory was used. The mix proposition of concrete is mentioned in Table 1. The maximum size of the coarse aggregate is 25 mm, the water–cement ratio is 41.5%, and the sand–aggregate ratio is 48.3%. The 28-day average design compressive strength of concrete is 35 MPa, measured based on KS F 2405 [19]. The 28-day compressive strength of the concrete was measured as 30.8 MPa.

#### 2.1.2. Reinforcement

**Figure 1:**
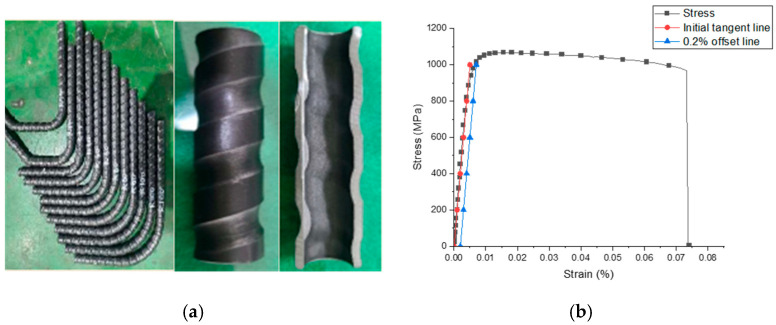
The photo and tensile stress–strain curve of tubular rebar: (**a**) tubular rebar photos; (**b**) stress–strain curve.

In this study, two types of steel rebars were used as reinforcement. SD400 modified steel rebar is classified as 19.1 mm-diameter rebar (D19) and 12.7 mm diameter rebar (D13), respectively, according to the nominal diameter [20]. D19 was used as longitudinal rebar, and D13 was used as transverse rebar and stirrup. Unlike the currently used deformed steel rebar, the newly developed tubular rebar is a steel pipe manufactured by rolling a high strength steel plate in a tube shape with rib. It has the same diameter as D19 but has half the cross-sectional area and twice the tensile strength [9]. This tubular rebar was manufactured by the Korean steel company POSCO (Pohang, Republic of Korea). The mechanical properties of the deformed rebar and tubular rebar are provided in Table 2. The tensile strength test of the tubular rebar induced the stress–strain curve of the 19.1 mm-diameter tubular rebar, which is provided in Figure 1. Since the yield point was not clearly distinguished in the stress–strain curve of the tubular rebar, the arbitrary yield point was calculated using the 0.2% offset method [21]. As a result of the offset evaluation, a yield strength of 1000 MPa was derived, and ultimate strain and ultimate strength were measured at 8.2% and 1069 MPa, respectively.

### 2.2. Specimen Design and Test Setup for Slab Specimen

A total of 4 slab specimens were fabricated to evaluate the structural performance when tubular rebar was applied to flexural members. When designing the structures used in this study, ACI 318-19 [13] was applied. The notation of each specimen is based on the nature of support, type of structure, type of reinforcement, and the number of main reinforcing bar. The first letter in the notation corresponds to the supporting system, either “P” for pinned supported or “F” for fully fixed. The second letter represents the type of structures, “S” for slab. The third letter is “D” for deformed rebar, and “T” for tubular rebar. The last number is the number of main rebars.

#### 2.2.1. Slab Specimen Design

The tubular rebar has a yield strength of 800 MPa or more, so it has not been confirmed that it is a material suitable for the current structural standard. However, the tubular rebar specimen was designed by applying the existing design formula to compare the bending performance with the deformed rebar.

**Figure 2:**
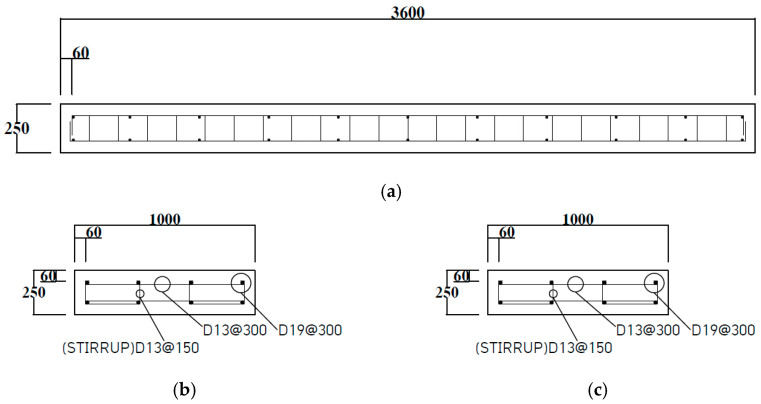

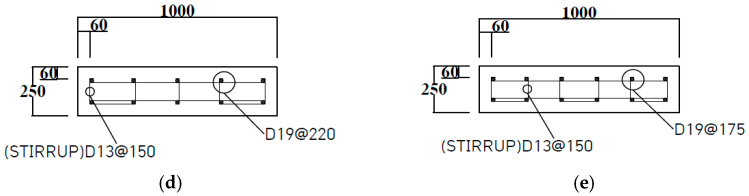
Slab specimens design details: (**a**) slab design details; (**b**) PS-D4; (**c**) PS-T4; (**d**) PS-T5; (**e**) PS-T6. (Unit: mm).

The specimens were designed with the amount of rebar as the main variables. In order to observe the difference according to the amount of tubular rebars, the number of main rebars was selected in three types: 4, 5, and 6, and double reinforcement was applied to observe the difference between the compression and tension behavior. Also, a specimen reinforced only with a deformed rebar was designed for comparison with tubular rebar. In order to prevent shear failure of the specimen, the stirrup assembled with D13 was placed, and the main rebar was designed to not more than the balanced reinforcement ratio to make it a low-reinforced slab. The specifications of the specimen are tabulated in Table 3. As shown in Figure 2, the design details of slab specimens were presented.

#### 2.2.2. Test Setup for 4-Point Bending

**Figure 3:**
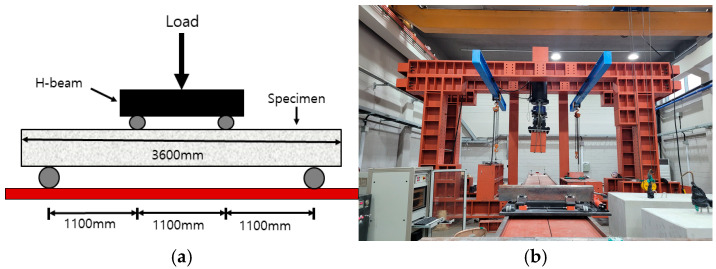
Test setup: (**a**) 4-point bending; (**b**) 2000 kN capacity actuator.

A 4-point bending test was carried out on the slab specimen [19]. The test setup detail for each specimen is shown in Figure 3. A hinge and roller were installed 150 mm away from both ends of each specimen for simple support. The loading point was set to 1100 mm for the slab specimen, which is 1/3 of the span. The load was applied in a displacement-controlled manner at a rate of 3 mm per minute, using a 2000 kN capacity actuator.

**Figure 4:**
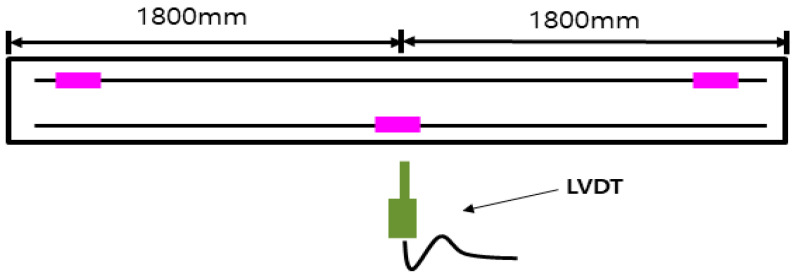
LVDT and strain gauges (in pink) location.

The strain gauges were attached to the reinforcement to measure the strain of the reinforcement according to the applied load. In addition, two linear variable displacement transducers (LVDT) with a capacity of 200 mm were installed at the lower end of the center of the specimen to measure the displacement of the member. Figure 4 showed the location and numbering of each measuring device.

### 2.3. Experimental Results of 4-Point Bending Test

#### 2.3.1. Failure Modes and Crack Behavior of 4-Point Bending Test

As a result of analyzing the crack behavior, the following similarities were observed in all specimens. A typical bending crack occurred in which cracks occurred in the vertical direction at the center of the slab, and the initial crack occurred in the lower part of the center of the specimen and progressed in a direction perpendicular to the upper part. As the load increased, it was confirmed that the crack spread from the center to both ends of the specimen.

**Figure 5:**
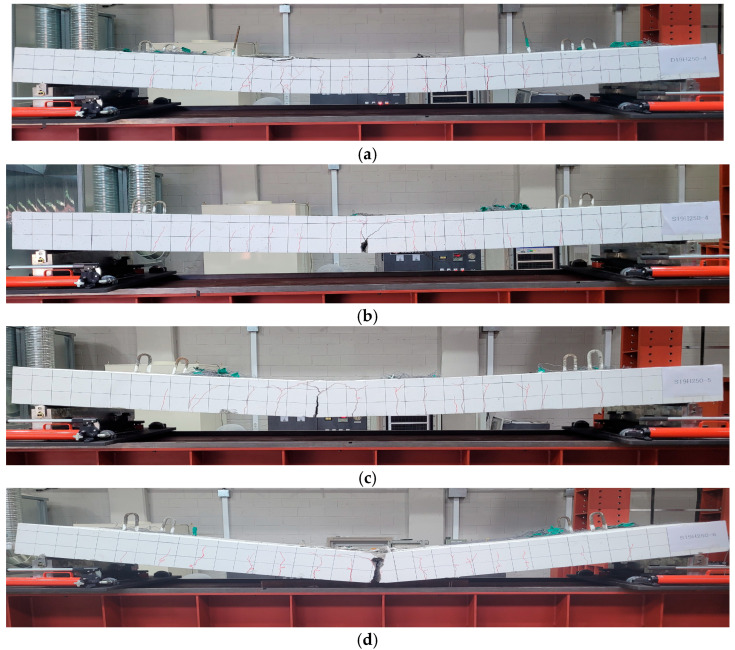
Failure mode and crack behavior for each specimen: (**a**) PS-D4; (**b**) PS-T4; (**c**) PS-T5; (**d**) PS-T6.

Significant differences were observed in the crack behavior of deformed rebar specimen and tubular rebar specimen. The deformed rebar specimen showed a ductile fracture pattern in which concrete crushing occurred at the top of the specimen after yield of the rebar. As expected, in the case of the tubular rebar specimen, it showed a larger crack than the deformed rebar specimen, and showed brittle fracture in which the member was rapidly broken at the same time as the yield of the rebar. As the number of main rebars increased, this pattern became more prominent, and more explosive fractures occurred. Figure 5 shows the overall crack pattern of each specimen.

#### 2.3.2. Load–Displacement Relationship of 4-Point Bending Test

**Figure 6:**
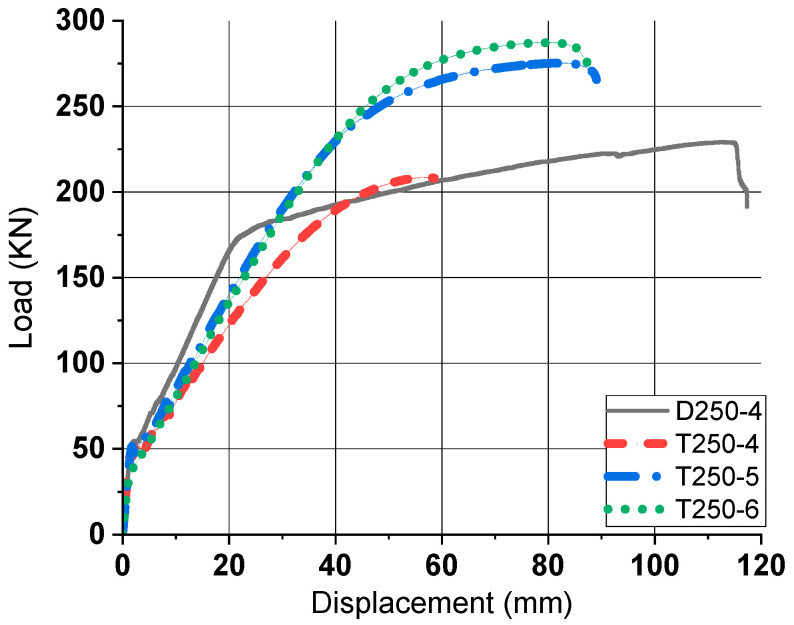
Experimental P–∆ relationship (pinned slab).

The load versus mid-span displacement curves (P–∆ curve) for all specimens are shown in Figure 6. In general, the P–∆ curve of the 1-way concrete slab is divided into three important points: initial crack point, yield point, and ultimate point. As shown in Fig.6, three points are clearly distinguished in the curves of PS-D4. However, in the tubular rebar specimens, the distinction between each point is ambiguous, and after reaching the ultimate strength point, a rapid rupture of the rebar appears, which can be confirmed by the rapid drop of the load. The tubular rebar specimens showed nonlinear behavior after initial crack occurred, so the position where the slope of each curve changed rapidly was set as the yield point. Table 4 summarizes the load and displacement values at the initial crack point (P_c_ and ∆_c_), yield point (P_y_ and ∆_y_) and ultimate point (P_u_ and ∆_u_) of each specimen. Comparing PS-D4 to PS-T4, with the same number of rebars, PS-T4 had 11.25% higher P_y_ and 9.25% lower P_u_ than PS-D4. In PS-T4, the excessive displacement increased by 87% and there was a rapid rupture after the ultimate point was reached. PS-T5 and PS-T6 exhibited 44.46% and 37.08% higher yield loads (P_y_), and 20.07% and 25.31% higher ultimate loads (P_u_), respectively, compared to PS-D4. Although these specimens demonstrated higher flexural strength, they also showed excessive displacement relative to PS-D4. Similar to PS-T4, a sudden rupture occurred shortly after reaching the ultimate load.

## 3. Numerical Evaluation of Slab Specimen

### 3.1. Numerical Analysis Procedure Using LS-DYNA

**Figure 7:**
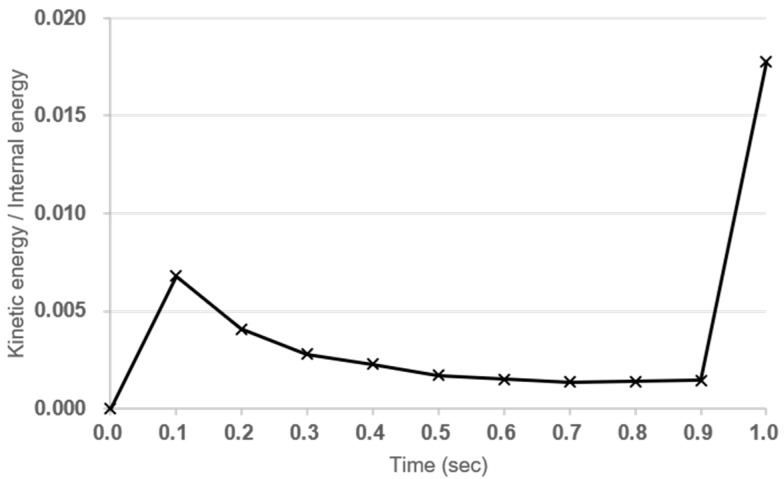
Time history of kinetic energy and internal energy during the simulation.

In this section, numerical analysis methods are presented to predict the bending performance of concrete structures reinforced with tubular rebars. LS-DYNA 970 [22], a commercial FEA program, was used for numerical analysis. This FEA program is used to analyze the nonlinear dynamic behavior of materials and structures. LS-DYNA provides time-dependent deflection by attributing Lagrangian formulation to Cartesian coordinate system. Momentum equation, traction boundary condition, displacement boundary conditions, and contact conditions were considered for structural behavior analysis. In this finite element analysis, the LS-DYNA explicit solver was used with a quasi-static approach. To suppress dynamic effects, a sufficiently slow displacement-controlled loading rate was applied, maintaining the kinetic energy below 5% of the internal energy throughout the simulation as shown in Figure 7 [23]. This is a commonly accepted criterion to ensure quasi-static conditions in explicit dynamic solvers. The loading rod was modeled as a rigid cylinder (MAT_RIGID), and vertical displacement was applied using BOUNDARY_PRESCRIBED_MOTION_RIGID to replicate the experimental actuator movement. The interaction between the rigid body and the concrete surface was defined using AUTOMATIC_SURFACE_TO_SURFACE contact. A displacement rate of 0.05 m/s was used to shorten simulation time, and it was verified through preliminary analysis that this did not affect the structural response compared to the experimental rate of 0.05 mm/s.

#### 3.1.1. Slab Specimen Modeling

**Figure 8:**
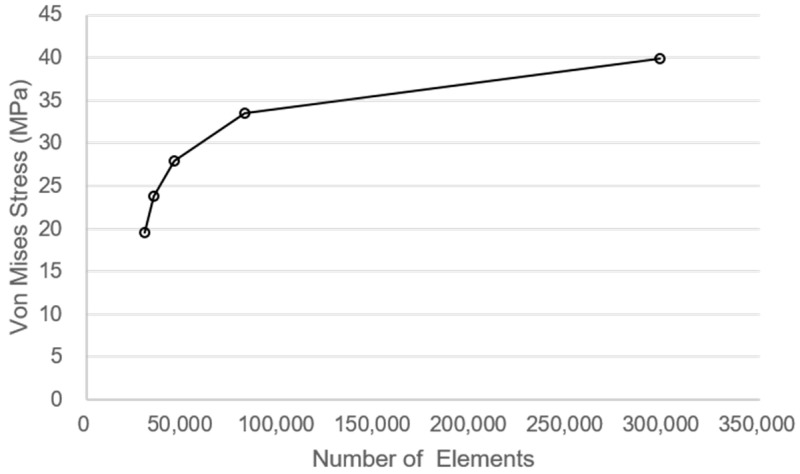
Mesh convergence analysis.

The concrete of slab specimen was modeled with solid element and rebar with beam element, and the loading rod was modeled with rigid element to implement the same conditions as the actual loading conditions. The hollow section of tubular rebar was implemented with the cross-section type of the beam elements as tubular. The size of one element of concrete was modeled to 25 mm, and the length of one element of rebar was modeled to 10 mm. A mesh convergence study was conducted to determine an appropriate element size for the concrete domain. As shown in Figure 8, the von Mises stress values stabilized as the number of elements increased, indicating convergence of the numerical solution. Considering both the computational efficiency and the maximum aggregate size of 25 mm used in the concrete mix, a mesh size of 25 mm (82,100 elements) was deemed appropriate for the analysis.

**Figure 9:**
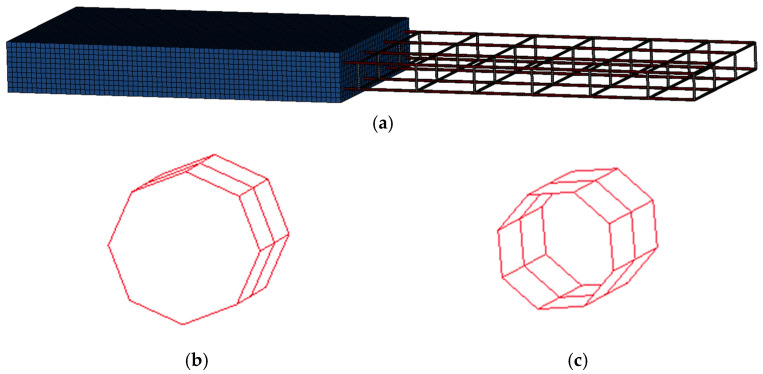
Finite elements model geometry: (**a**) slab specimen model geometry; (**b**) deformed rebar cross section; (**c**) tubular rebar cross section.

Figure 9 showed the cross-sectional shape of rebar and FE model geometry. In bending structure design, it is basically assumed that the rebar and concrete are fully bonded [13]. Therefore, the interaction between the beam elements (used to model both deformed and tubular rebars) and the solid concrete elements was defined using the CONSTRAINED_LAGRANGE_IN_SOLID (CLIS) keyword in LS-DYNA. This approach allows for independent meshing of the reinforcement and concrete, facilitating modeling of complex geometries without the need for coincident nodes. The CLIS method enforces a perfect bond between the reinforcement and concrete, assuming no relative slip at the interface. This assumption aligns with the experimental observations, where no significant slip or debonding was noted prior to peak load.

The suitability of the CLIS approach for modeling reinforced concrete structures has been validated in previous studies. For instance, Moutoussamy et al. (2011) demonstrated that CLIS, combined with the continuous surface cap model (CSCM) for concrete, effectively captures the behavior of reinforced concrete beams and frames under pseudo-dynamic loading conditions, accurately representing moment transmission and plastic hinge formation [24]. Furthermore, Tay et al. (2016) investigated the effectiveness of CLIS in coupling beam elements to concrete in ALE simulations, confirming its applicability in scenarios involving complex interactions between reinforcement and concrete [25].

In FE model, the mesh size of the solid concrete elements was set to 25 mm, while the beam elements representing the reinforcement had a length of 10 mm. This configuration ensured that each rebar element was embedded in at least two to three surrounding concrete elements, satisfying the mesh compatibility criteria recommended for CONSTRAINED_LAGRANGE_IN_SOLID coupling in LS-DYNA [22].

**Figure 10:**
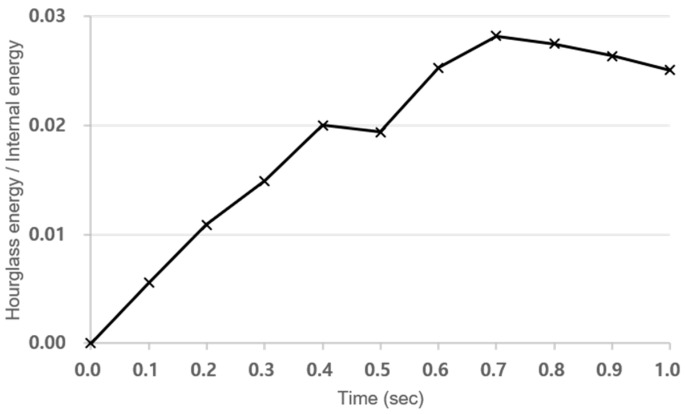
Time history of hourglass energy and internal energy during the simulation.

The specimen’s support conditions were implemented by assigning a single point constraint (SPC). In addition, the automatic surface-to-surface command prevented the loading rod from penetrating into the solid element of concrete. To prevent hourglass deformation in reduced integration elements, the viscous hourglass control method was applied with a coefficient of 0.05. During the simulation, hourglass energy remained below 5% of the internal energy as shown in Figure 10, which satisfies a commonly accepted criterion for ensuring numerical stability in explicit analysis. To simulate damping effects, stiffness-proportional damping was applied using the DAMPING_GLOBAL keyword with a coefficient value of 0.05, based on preliminary calibration to match the experimentally observed damping behavior. The damping parameters were carefully selected to avoid artificially suppressing the dynamic response and were verified not to affect the overall behavior of the system.

#### 3.1.2. Material Modeling

In this study, the CSCM provided by the LS-DYNA program was selected as the concrete material model. This model is built on three input specifications: the unconfirmed compression strength, the aggregate size, and the unit. CSCM is also valid for normal compressive strength of 28 MPa to 58 MPa [26].

Rebar was modeled with a PIECEWISE LINEAR PLASTICITY material model (MAT024). This material model is based on the input specifications of mass density, Young’s modulus, Poisson’s ratio, yield strength, and tangent modulus. In general, steel rebars are simply described as having bilinear stress–strain relationships. However, in this FE model, MAT024 was used to show the stress–strain relationship of the tubular rebar to have a multi-linear relationship.

### 3.2. Numerical Results

#### 3.2.1. Failure Modes and Crack Behavior Observed in FEA

**Figure 11:**
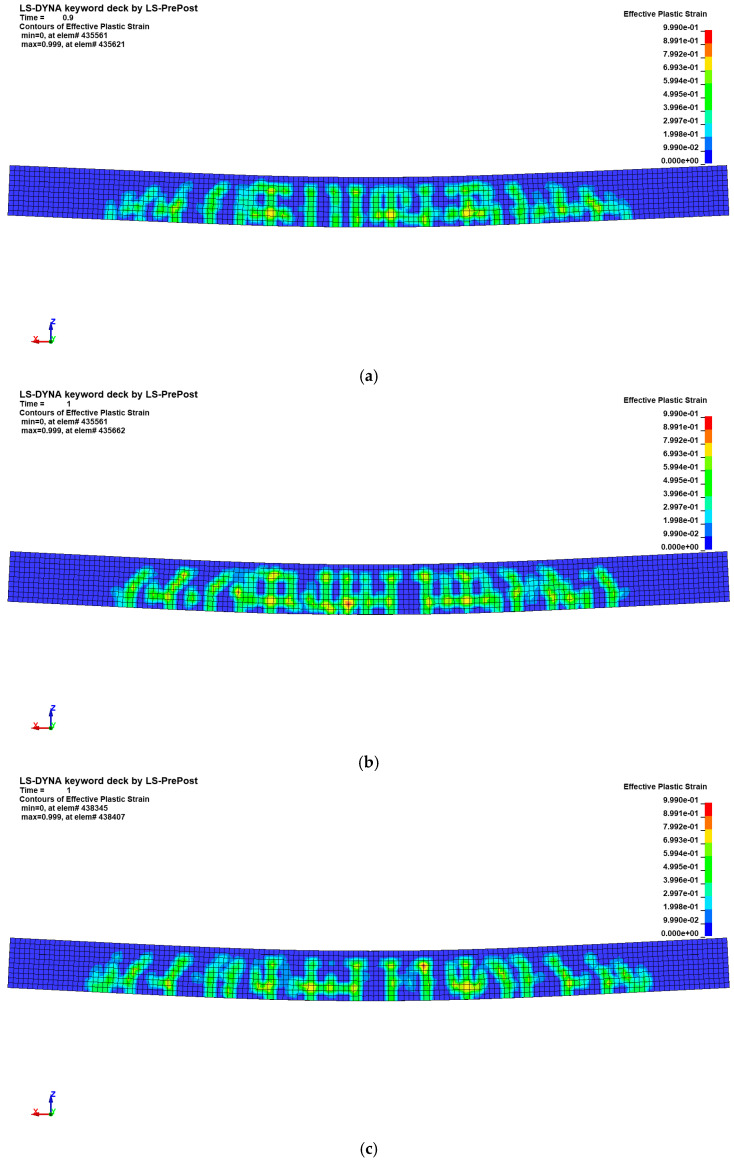

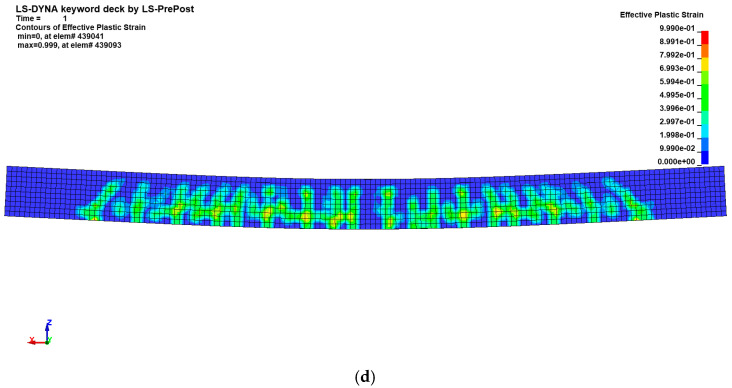
Failure mode and crack behavior from numerical results (effective plastic strain): (**a**) PS-D4; (**b**) PS-T4; (**c**) PS-T5; (**d**) PS-T6.

Figure 11 depicts the modes of failure and crack behavior for slab specimen as observed from FEA. Although the CSCM model did not capture the complete brittle failure observed in the experiment for tubular-reinforced specimens, it successfully reproduced the initial crack formation and propagation up to the peak load. This reflects the quasi-brittle behavior of concrete prior to sudden strength degradation. As with the experimental results, the initial crack occurred in the lower part of the center of the specimen and progressed in a direction perpendicular to the upper part. The range to which cracks occur was also predicted similar to the experimental results. In addition, strain is distributed in a similar pattern to the cracks observed in Figure 5.

In this simulation, erosion was activated in the CSCM concrete model using an effective plastic strain threshold of 1.1. This relatively high threshold prevented premature element deletion but also limited the model’s ability to reproduce the post-peak brittle fracture observed in the experiment. As noted in the FHWA technical report (FHWA-HRT-05-063), the CSCM model can simulate quasi-brittle behavior when properly calibrated with appropriate erosion and damage evolution parameters [26]. In future work, these settings will be refined to enhance the model’s ability to capture post-peak localization and fracture in reinforced concrete systems.

#### 3.2.2. Load–Displacement Relationship and Ductility

**Figure 12:**
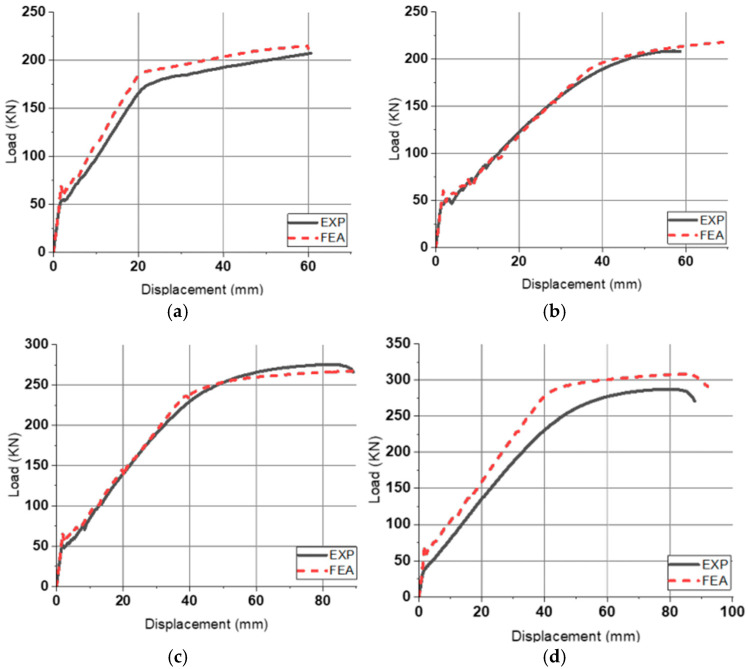
P–∆ comparison for pinned slab: (**a**) PS-D4; (**b**) PS-T4; (**c**) PS-T5; (**d**) PS-T6.

The P–∆ curves derived from experiments and FEA conducted on slab specimen were compared. Figure 12 depicts this comparison. The three points of initial crack point, yield point, and ultimate point were determined in the same way as in the experiment. Similar to the experimental results, the behavior of tubular rebar specimens was nonlinear after initial crack occurred. Based on the FE results, the error rate of the experimental results and the FEA results were compared. The bending performance in the FE model had an average difference of 5.01% compared to the experiments. In addition, when comparing the slope of the linear stage between the initial crack point and the yield point, experiments and FEA have a difference of 1.22% on average. Therefore, the prediction of bending behavior of tubular rebars using FEA has a small error compared to the results of the experiment. In this study, two methods were used to evaluate ductility: displacement ductility method and energy ductility method. The displacement ductility (µ_∆_) is defined as the ratio of yield displacement (∆_y_) to ultimate displacement (∆_u_). In Equation (1), it was proposed by Naaman and Jeong [27] as a formula for the energy ductility index (µ_E_). E_t_ is the total energy, the total area of the P–∆ curve. E_e_ is elastic energy, which is deformation energy stored without being consumed in the total energy.

(1) $$\mu_{E}=2\left(\frac{E_{t}}{E_{e}}+1\right)$$

Table 5 summarizes the FE results and ductility of pinned slabs. On average, the tubular rebar specimens demonstrated 22.48% lower µ_∆_ and 27.79% lower μ_E_ compared to PS-D4. Compared to PS-D4, all tubular rebar members had higher yield load and ultimate load. However, the specimens using tubular rebars have excessive displacement compared to the deformed rebar specimens. Therefore, it is concluded that the use of tubular rebars requires limitation of flexural displacement, and to verify this, the bending behavior in the statically indeterminate state should be simulated.

### 3.3. Fixed Slab Model

In the previous sections, the bending behavior of simply supported slabs using tubular rebars demonstrated brittle failure characteristics due to insufficient ductility. This limitation was primarily attributed to the geometry of the tubular rebar and the absence of internal moment redistribution in the statically determinate system. To address this, the model was optimized by changing the boundary conditions to fixed ends, resulting in a statically indeterminate slab model.

The objective of this optimized model was to investigate whether ductility could be improved through structural configuration and internal force redistribution, rather than solely relying on material properties. By adopting fixed-end conditions, the structure was able to resist displacement more effectively and redistribute stresses, potentially mitigating the early brittle failure observed in the simply supported configuration. This change in modeling approach reflects a realistic application scenario and is intended to verify the feasibility of tubular rebar in practical structural systems.

The fixed slab models were analyzed with varying reinforcement ratios to compare their performance with those using conventional deformed rebars. The results are presented in the following figures and discussed in terms of load–displacement behavior, failure patterns, and ductility enhancement.

**Figure 13:**
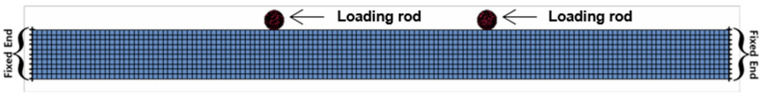
Boundary condition detail for fixed slab.

Previous experiments and FEA confirmed that members using tubular rebar have high bending strength despite having low reinforcing ratio, but have low curvature ductility. Therefore, it has been confirmed that securing ductility is essential to use tubular rebar as bending members. To verify this, the slab, which was simply supported in the previous section, was fixed at both ends to be statically indeterminate, and the bending performance in this state was analyzed. In this section, the previous pinned slab was remodeled into a fixed condition. The material model and model geometry used for modeling are the same as those presented in Section 3.1.2. Figure 13 describes the boundary condition of the FE model.

**Figure 14:**
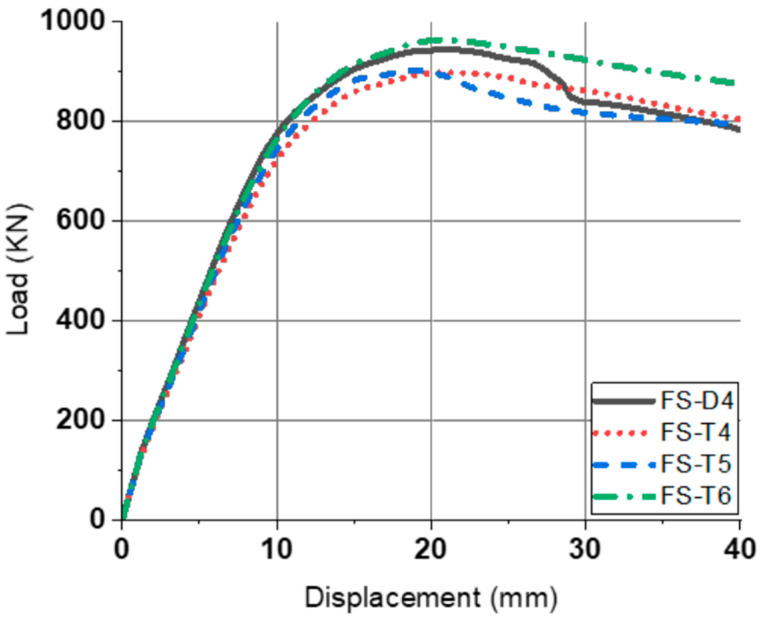
P–∆ relationship (fixed slab).

In the fixed slab, no clear initial crack was observed, and linear elastic behavior was performed before the occurrence of the yield. Crack initiation primarily occurred in the mid-span tension zone of the slab and propagated upward with increasing load. Due to the fixed-end condition, stress redistribution toward the support regions was observed, delaying brittle failure and enhancing ductility. The failure was dominated by flexural behavior. Therefore, the yield point, ultimate point, and ductility index are described in Table 6 and Figure 14. It was confirmed that all specimens have a higher yield load and ultimate load than pinned slabs. In the case of fixed slabs, tubular rebar specimen has, on average, 0.28% lower $\mu_{∆}$ and 3.96% lower $\mu_{E}$. Compared to the ductility of the pinned slab presented in Table 5, the difference between the ductility of the tubular rebar and the deformed rebar was greatly reduced. The FS-T6 has a higher ductility and a higher ultimate load compared to the FS-D4. This result showed that tubular rebar has improved bending performance at a lower rebar ratio when used in statically indeterminate structures.

## 4. Experimental Evaluation of Culvert Specimen

### 4.1. Specimen Design and Test Setup of Culvert Specimen

In the previous section, the bending behavior of members applied with tubular rebar was analyzed numerically in two states: statistically determinate and statically indeterminate. Therefore, in this section, an experimental verification was performed by performing a bending test on a box culvert, a representative statically indeterminate structure.

#### 4.1.1. Culvert Specimen Design

**Figure 15:**
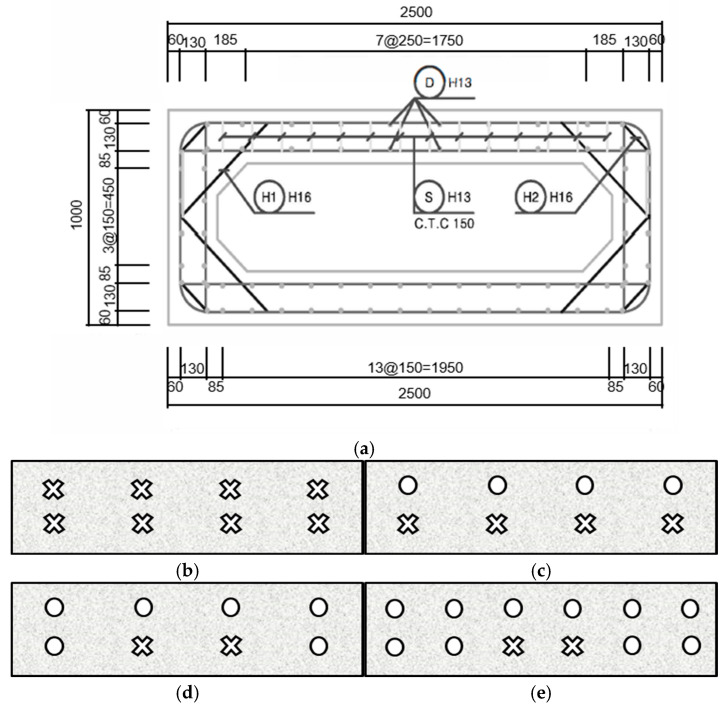
Culvert specimens design details: (**a**) standard cross-section; (**b**) C-DD4; (**c**) C-TD4; (**d**) C-TH4; (**e**) C-TH6. X: deformed rebar, O: tubular rebar.

In Section 4, culvert specimens were tested. For comparison, a control specimen using only the deformed rebar was produced, along with a specimen that replaced only the compression part with the tubular rebar, a specimen that replaced the tensile part with the deformed and tubular hybrid, and a specimen that increased the rebar ratio. The notation of each specimen is based on the type of structure, type of compression rebar, type of tensile rebar, and the number of main reinforcing bar. The first letter “C” corresponds to the type of structure: culvert. The second and third letter represent the type of rebar in the compressive and tensile part, respectively, “D” for deformed rebar, “T” for tubular rebar, and “H” for hybrid. The last number is the number of main rebars. In addition, Table 7 and Figure 15 showed differences in the arrangement design of each culvert specimen.

#### 4.1.2. Test Setup for 3-Point Bending

**Figure 16:**
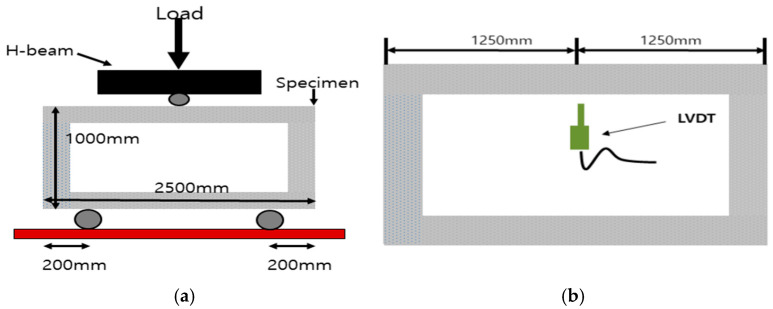
Test setup of culvert specimen: (**a**) 3-point bending; (**b**) LVDT position.

For culvert specimens, a 3-point bending test was carried out [19]. The test setup detail for each specimen is shown in Figure 16. The hinge and roller were installed at 200 mm away from both ends of each specimen. The load was applied the same way in slab specimens.

### 4.2. Experimental Results of 3-Point Bending Test

#### 4.2.1. Failure Modes and Crack Behavior of 3-Point Bending Test

**Figure 17:**
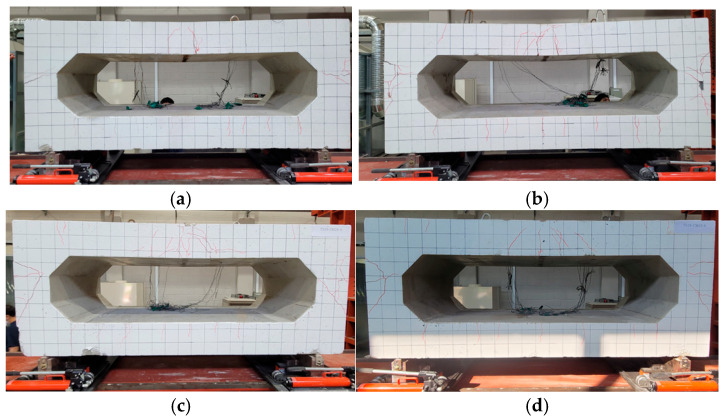
Crack details in culvert specimen: (**a**) C-DD4; (**b**) C-TD4; (**c**) C-TH4; (**d**) C-TH6.

The front and side cracks of the culvert appear in Figure 17. As a result of the experiment, typical bending cracks appear at the top. The initial crack occurred below the center and progressed gradually in the vertical direction. In the culvert specimen, the vertical load applied to the top slab was transferred through the sidewalls to the foundation. Due to bending of the top slab and restraint at the sidewalls, flexural tension cracks first developed at the bottom surface near mid-span. As the load increased, diagonal cracks propagated from the corners (haunch regions), indicating a combination of flexural and shear failure. Stress concentration was observed along the internal corners, which ultimately governed the failure pattern. The fixed boundary condition provided by the box geometry contributed to stress redistribution, which delayed localized failure and enhanced the overall load-carrying capacity.

#### 4.2.2. Load–Displacement Relationship of 3-Point Bending Test

**Figure 18:**
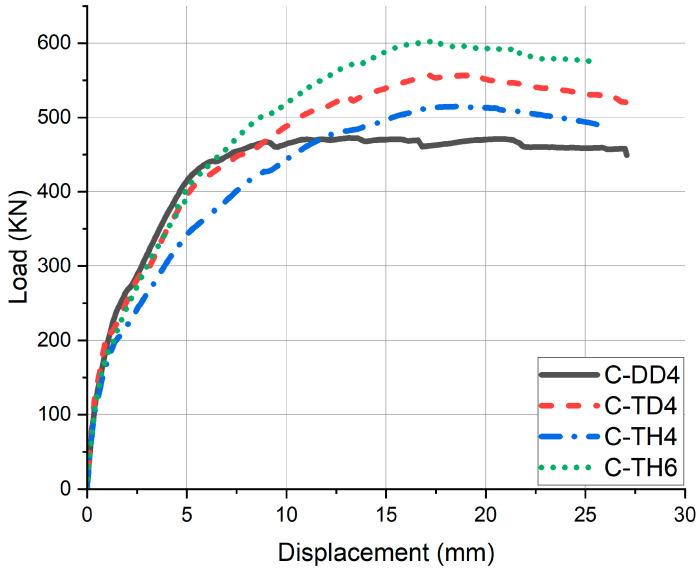
Experimental P–∆ relationship (culvert).

The experimental results were showed in Figure 18 and Table 8. The three point of the initial crack point, yield point, and ultimate point were determined in the same way as in the slab specimen. C-DD4 displayed a typical bi-linear response. From the initial crack point to the yield point, linear elastic behavior was shown, and then the displacement increased without increasing the load. C-TD4 had 12.01% higher P_c_, 3.37% higher P_y_, and 17.85% higher P_u_ than C-DD4. Compared to C-DD4, it is confirmed to have similar stiffness and greater strain energy. C-TH4 had 1.83% higher P_c_, 3.12% higher P_y_, and 8.96% higher P_u_ than C-DD4. As shown in the Figure 11, replacing the tensile reinforcement increased the ultimate load but increased the displacement to yield. C-TH6 had 9.15% lower P_c_, 23.29% higher P_y_, and 27.51% higher P_u_ than C-DD4. The results showed the high flexural strength of C-TH6 and confirmed its effective replacement of tubular rebar.

## 5. Discussion

### 5.1. Demands for Statically Indeterminate States

Through the previous experiment and FEA, the bending performance of pinned slab, fixed slab, and box culvert using tubular rebar was reviewed. In the case of pinned slab, tubular rebars, like general high strength rebars, had high yield strength and extreme strength, but showed excessive displacement of members due to low ductility. Therefore, it was confirmed that the displacement of members should be limited in the use of tubular rebars. FS-T5, which had a reinforcing ratio of 62.5% compared to FS-D4, had almost the same ductility before the yield of the member, but has a low ultimate load, and FS-T6, which has a reinforcing ratio of 75% shows a similar ductility and a higher ultimate load. Through this, it was verified that tubular rebars can have improved bending performance at a lower reinforcing ratio in a statically indeterminate state.

The experimental observations provide valuable insights into how the geometry of reinforcement influences failure mechanisms. This also validates the need for accurate material modeling to capture such effects, as demonstrated in the numerical simulation.

### 5.2. Effect of Replacing Reinforcement

In order for tubular rebars to be effectively used as bending members by replacing deformed rebars, the structure should be in a statistically indeterminate state with restrictions on displacement. In that state, as shown in results of the fixed slab and box culverts, it should have a reinforcement ratio of 75% or more compared to deformed rebar members. As can be seen in the case of C-TD4, in the case of replacing the compression part, a very effective bending rebar replacement performance was confirmed. In the case of the tensile part, as observed in the case of C-TH4, it may have a high bending strength, but the amount of displacement increases. Therefore, like C-TH6, when tubular rebars and deformed rebars are fabricated as hybrid and the rebar ratio is 87.5% or higher, similar ductility can be observed as well as higher bending strength.

The experimental results also revealed that tubular rebar specimens tended to fail in a brittle manner, particularly in statically determinate conditions. This behavior was attributed to stress concentration at the hollow section, which accelerated localized fractures. These observations emphasize that while tubular rebars may limit ductility, they can be advantageous in statically indeterminate systems where internal force redistribution is allowed. The experimental insights thus reinforce the importance of structural configuration when considering tubular reinforcement.

While this study focuses primarily on the structural performance and material efficiency of tubular rebars, it is important to note that a complete sustainability assessment should also consider energy consumption in the manufacturing process, transport, and construction, as well as life-cycle environmental impacts. Future research is planned to evaluate the embodied energy and carbon footprint associated with tubular reinforcement to provide a more comprehensive understanding of its sustainability potential.

## 6. Conclusions

Based on the experimental and numerical results conducted in this study, the conclusions are as follows:

1. Tubular rebar has high bending strength when used as a flexural reinforcement, but in statically determinate structures such as pinned slabs, its low ductility can lead to sudden failure of the member. This low ductility, as observed in the P–Δ curve of the tubular rebar specimen, is primarily caused by stress concentration around the hollow section, which produces approximately 73.20% higher stress compared to conventional deformed rebar. Therefore, tubular rebar can exhibit effective flexural performance when applied to statically indeterminate structures that are capable of limiting displacement.
2. Tubular rebar has a high substitution efficiency in the compression part. When all compression rebars were replaced with tubular rebars, the same ductility and high bending strength are observed. However, when tensile rebars were replaced, a substitution efficiency was observed only at a reinforcing ratio of 82.5% or more.
3. The tubular rebar is a high strength rebar with high bending performance and is an efficient material for reducing GHG emissions by using less raw materials. However, due to low ductility, rapid rupture occurs after yield in a simply supported slab. Therefore, it is expected to show efficient rebar replacement performance if it is designed with a limit on maximum deflection when applying tubular rebar.
4. Compared to conventional deformed rebar specimens, tubular rebar specimens exhibited a yield load increase of up to 44.46% (PS-T5 vs. PS-D4) and an ultimate load increase of up to 25.31% (PS-T6 vs. PS-D4) under the same slab configuration. However, the displacement ductility index was reduced by up to 35.6%, confirming the need for displacement control. In statically indeterminate structures, such as fixed slabs, the difference in ductility index between tubular and deformed rebars was reduced to less than 3%, and in some cases (FS-T6), tubular rebars even exceeded the ductility and strength of deformed rebars.

## Figures and Tables

**Table 1:** Mix proportion of concrete (by weight).

Cement	Water	Fine Aggregate	Coarse Aggregate	Admixture
1.0000	0.4147	1.9336	2.1635	0.0075

**Table 2:** Mechanical properties of steel rebars.

Notation	Yield Strength (MPa)	Tensile Strength (MPa)	Elongation Rate (%)	Elastic Modulus (MPa)
Deformed rebar	400~520	≥460	≥16	200,000
Tubular rebar	≥800	≥860	≥10	

**Table 3:** Specification of slab specimens.

Specimen ID	Steel Rebar Type	Dimension (mm)	Reinforcement Ratio	Spacing (mm)
PS-D4	Deformed	250 × 1000 × 3600	0.012693 (100%)	300
PS-T4	Deformed		0.006351 (50.0%)	300
PS-T5	Tubular		0.007939 (62.5%)	220
PS-T6	Tubular		0.009526 (75.0%)	175

**Table 4:** Experimental results of pinned slab specimens.

Specimen	Initial Crack Point		Yield Point		Ultimate Point	
	P_c_ (kN)	∆_c_ (mm)	P_y_ (kN)	∆_y_ (mm)	P_u_ (kN)	∆_u_ (mm)
PS-D4	54.4	2.18	161.54	19.25	229.2	112.3
PS-T4	37.0	1.01	179.72	35.98	208.0	58.59
PS-T5	50.1	2.87	233.36	41.17	275.2	82.89
PS-T6	44.5	3.01	221.44	37.62	287.2	78.63

**Table 5:** Numerical results of pinned slab specimens.

Specimen	Initial Crack Point		Yield Point		Ultimate Point		Ductility Index	
	P_c_ (kN)	∆_c_ (mm)	P_y_ (kN)	∆_y_ (mm)	P_u_ (kN)	∆_u_ (mm)	µ_∆_	µ_E_
PS-D4	54.4	2.18	161.54	19.25	229.2	112.3	2.98	4.70
PS-T4	37.0	1.01	179.72	35.98	208.0	58.59	1.92	2.52
PS-T5	50.1	2.87	233.36	41.17	275.2	82.89	3.03	4.28
PS-T6	44.5	3.01	221.44	37.62	287.2	78.63	1.99	3.39

**Table 6:** Numerical results of fixed slab specimens.

Specimen	Yield Point		Ultimate Point		Ductility Index	
	P_y_ (kN)	∆_y_ (mm)	P_u_ (kN)	∆_u_ (mm)	µ_∆_	µ_E_
PS-D4	678.84	8.21	943.60	20.61	2.51	1.78
PS-T4	625.83	8.19	898.28	20.86	2.55	1.75
PS-T5	638.91	8.07	902.78	18.93	2.35	1.64
PS-T6	650.05	7.98	963.18	20.88	2.62	1.73

**Table 7:** Specification of culvert specimens.

Specimen	Compression Part	Tensile Part	Reinforcing Ratio
C-DD4	Deformed rebar	Deformed rebar	0.012693 (100%)
C-TD4	Tubular rebar	Deformed rebar	0.009522 (75%)
C-TH4	Tubular rebar	Hybrid	0.007937 (62.5%)
C-TH6	Tubular rebar	Hybrid	0.11112 (87.5%)

**Table 8:** Experimental results of culvert specimens.

Specimen	Initial Crack Point		Yield Point		Ultimate Point	
	P_c_ (kN)	∆_c_ (mm)	P_y_ (kN)	∆_y_ (mm)	P_u_ (kN)	∆_u_ (mm)
C-DD4	189.54	0.96	409.62	4.86	472.42	13.17
C-TD4	212.30	1.26	423.44	4.49	556.76	19.08
C-TH4	193.00	1.31	422.40	8.54	514.76	18.50
C-TH6	172.20	0.90	505.00	9.18	602.4	17.34

## Data Availability

The raw data supporting the conclusions of this article will be made available by the authors on request.
